# CLEC12A signaling represses protective immune responses and contributes to hippocampal pathology in neurotropic picornavirus infection

**DOI:** 10.1038/s41598-025-27365-3

**Published:** 2025-11-10

**Authors:** M. K. Ameen, M. Stoff, S. Pavasutthipaisit, T. Ebbecke, M. Ciurkiewicz, T. Störk, J. Ruland, B. Lepenies, A. Beineke

**Affiliations:** 1 Department of Pathology, University of Veterinary Medicine Hannover, Hannover, Germany; 2 Center for Systems Neuroscience, Hannover, Germany; 3 Institute for Immunology, University of Veterinary Medicine Hannover, Hannover, Germany; 4 Research Center for Emerging Infections and Zoonoses, University of Veterinary Medicine Hannover, Hannover, Germany; 5 Institute of Clinical Chemistry and Pathobiochemistry, School of Medicine, Technical University of Munich, 80333 Munich, Germany; 6 Center for Translational Cancer Research (TranslaTUM), 81675 Munich, Germany; 7 German Cancer Consortium (DKTK), Partner Site Munich, 80336 Munich, Germany; 8 German Research Center (DKFZ), 69120 Heidelberg, Germany; 9 German Center for Infection Research (DZIF), Partner Site Munich, 17493 Greifswald, Germany; 10 Chair of Biochemistry and Chemistry, Veterinary Faculty, Ludwig-Maximilians-Universität München, Munich, Germany

**Keywords:** CLEC12A, TMEV, Neuroinflammation, Hippocampal pathology, Antiviral immunity, C-type lectin receptor, Immunology, Microbiology, Neurology, Neuroscience

## Abstract

Neurotropic viruses infect the central nervous system (CNS) and can cause severe neurological disorders. Theiler’s murine encephalomyelitis virus (TMEV) infection in C57BL/6 mice serves as a model for virus-induced encephalitis and hippocampal damage. C-type lectin domain family 12 member A (CLEC12A) is an inhibitory receptor, which modulates immune responses during inflammatory processes. However, the role of CLEC12A during neurotropic virus infections remains unclear. In this study, CLEC12A-deficient (CLEC12A^−/−^) and wild type C57BL/6 mice were infected with TMEV. Neuroinflammatory responses, viral load, and immune cell infiltration were analyzed using immunohistochemistry, RT-qPCR, and flow cytometry. CLEC12A^−/−^ mice exhibited increased T cell sequestration in the brain, along with a higher expression of pro-inflammatory cytokine (*TNF-α*, *IL-1β*) and antigen presentation genes (*CD11c*, *CD80*, *MHC-I*) during acute infection. This led to an improved viral clearance in the hippocampus. CLEC12A deficiency also activates splenic CD4^+^ T cells and CD8^+^ cytotoxic T cells upon infection. Despite increased peripheral T cell activation and neuroinflammation, CLEC12A^−/−^ mice displayed less hippocampal damage with improved neuronal and axonal integrity. In conclusion, CLEC12A signaling in C57BL/6 mice contributes to suppressive immune modulation, delaying viral elimination and exacerbating brain damage during acute neurotropic virus infection.

**Supplementary Information:**

The online version contains supplementary material available at 10.1038/s41598-025-27365-3.

## Introduction

Neurotropic viruses have the ability to infect the central nervous system (CNS) and cause neurologic disorders in humans and animals. Although most viral infections are asymptomatic and self-limiting in immunocompetent hosts, some can lead to fatal or debilitating disease. Moreover, hippocampal damage accompanied by long-term cognitive deficits and an increased risk of developing epilepsy can be observed in survivors of acute viral encephalitis^1–3^. Besides viral properties, CNS lesion development and disease outcome also depend on host immune responses. Here, early pathogen sensing by innate immune cells and the timely onset of protective antiviral immunity are crucial for viral elimination, but uncontrolled responses can cause immune-mediated brain injury in neurotropic virus infections^4,5^.

Theiler’s murine encephalomyelitis virus (TMEV) is a neurotropic picornavirus that primarily targets the hippocampus of mice^6–9^. While TMEV infection of SJL mice results in viral persistence, C57BL/6 mice rapidly eliminate the virus following acute encephalitis^10,11^. However, resulting robust inflammatory responses in C57BL/6 mice also lead to hippocampal damage, which manifests as cognitive deficits, impaired spatial memory and seizure development, making TMEV infection a well-established model for viral encephalitis-associated neurodegeneration^7,10,12–14^. Particularly, the early activation of innate immune cells, including CNS-infiltrating macrophages and microglia, triggers antiviral T cell responses in C57BL/6 mice, but significantly contributes also to neuropathology following TMEV infection by releasing neurotoxic factors^6,10,11,15–17^.

Myeloid C-type lectin receptors (CLRs) are pattern recognition receptors that recognize glycan structures on pathogen- or damage-associated molecular patterns in inflammatory disorders^18–20^. Activating CLRs utilize immunoreceptor tyrosine-based activation motifs (ITAM) and activate the spleen tyrosine kinase (Syk) for innate immune cell stimulation. In contrast, inhibitory CLRs contain an immunoreceptor tyrosine-based inhibitory motif (ITIM), which recruits tyrosine phosphatases and counteracts Syk to negatively modulate cellular activation^21–28^. Previous studies revealed that the inhibitory myeloid CLR dendritic cell immunoreceptor (DCIR) contributes to acute hippocampal injury by reducing antiviral immunity in C57BL/6 mice upon TMEV infection, indicating that inhibitory CLRs represent potential targets for intervention strategies to selectively enhance protective immunity in neurotropic virus infection^29^.

C-type lectin domain family 12 member A (CLEC12A; also known as myeloid inhibitory C-type lectin receptor, MICL) is an inhibitory CLR expressed on innate immune cells, including macrophages, monocytes and dendritic cells^30,31^. CLEC12A signaling negatively regulates immune responses and has been shown to suppress host immune responses in mice following mycobacterial infection^32^. In addition, it limits sterile inflammation and ameliorates immunopathology in murine colitis and rheumatoid arthritis models^33–35^. In contrast, CLEC12A signaling contributes to neuropathology in cerebral malaria models and experimental autoimmune encephalitis, demonstrating a complex and likely disease context-dependent function of the CLR in inflammatory disorders^36,37^. Interestingly, CLEC12A has been shown to promote protective antiviral immunity in lymphocytic choriomeningitis virus (LCMV) and influenza virus infection^38^. However, whether this represents a universal effect in viral disorders and whether CLEC12A signaling influences brain pathology in neurotropic virus infections remains undetermined.

The study aims to characterize the effect of CLEC12A signaling on virus control and neuropathology in TMEV infection. The findings indicate that intact CLEC12A signaling in C57BL/6 mice contributes to antiviral immunity, while CLEC12A deficiency is associated with enhanced peripheral T cell responses and leukocyte recruitment to the brain, thereby preventing hippocampal injury following neurotropic virus infection.

## Results

### CLEC12A deficiency leads to enhanced inflammation and reduced viral load in the brain

**Fig. 1:**
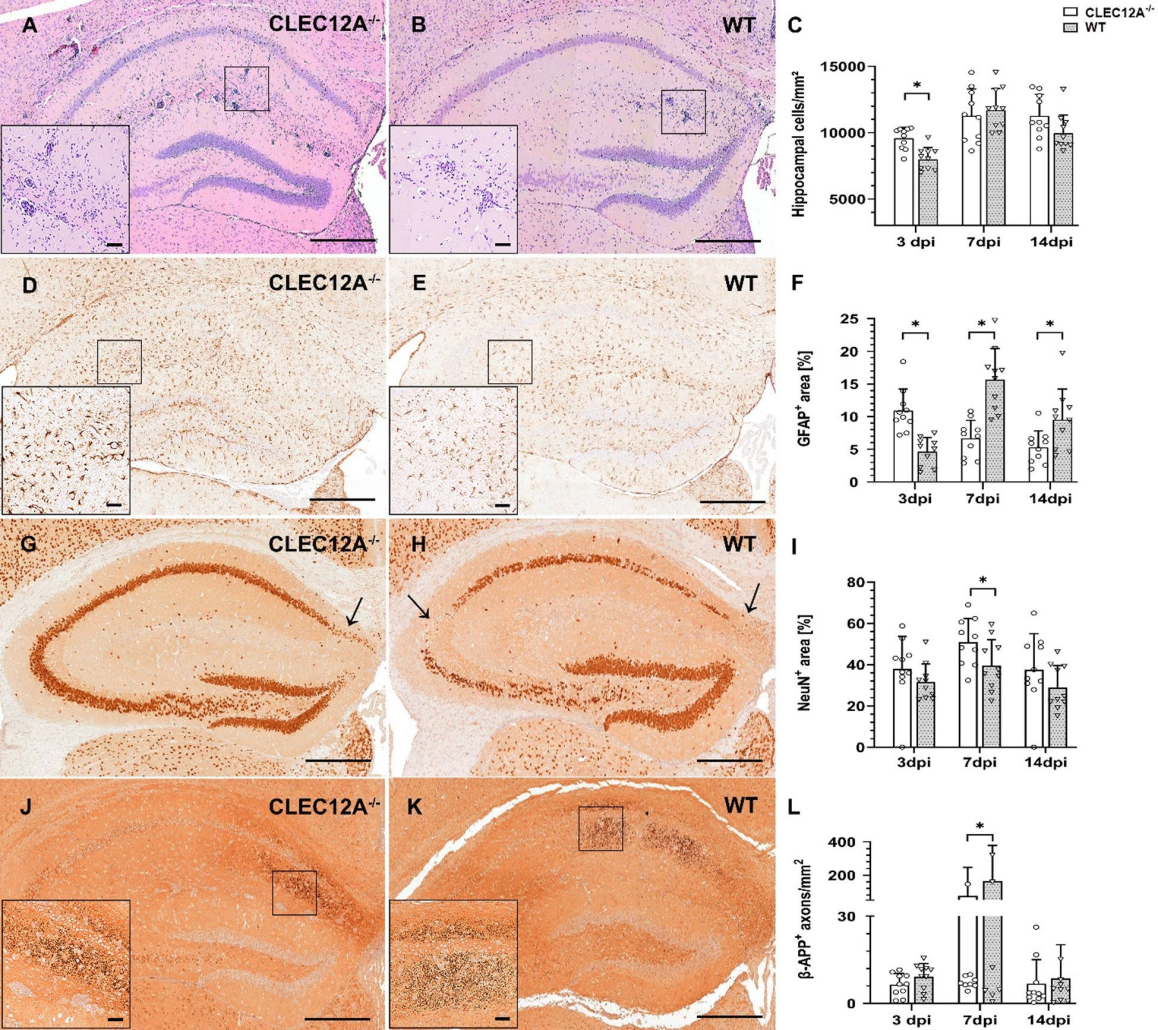
Neuropathology in CLEC12A^−/−^ mice and C57BL/6 wild type (WT) mice following Theiler’s murine encephalomyelitis virus (TMEV) infection. (**A–C**) Enhanced infiltration of inflammatory cells in CLEC12A^−/−^ mice. Representative images (histology) showing increased mononuclear cell infiltration in the hippocampus of (**A**) CLEC12A^−/−^ mice compared to (**B**) WT mice at 3 days post infection (dpi). (**D–F**) Enhanced astrogliosis in CLEC12A^−/−^ mice. Representative images (immunohistochemistry) showing increased numbers of GFAP^+^ astrocytes in (**D**) CLEC12A^−/−^ mice compared to (**E**) WT mice at 3 dpi. (**G–I**) Reduced neuronal loss in CLEC12A^−/−^ mice. Arrows in representative images (immunohistochemistry) showing more NeuN^+^ pyramidal neurons in (**G**) CLEC12A^−/−^ mice compared to (**H**) WT mice at 7 dpi. (**J–L**) Decreased axonal damage in CLEC12A^−/−^ mice. Representative images (immunohistochemistry) showing reduced numbers of β-APP^+^ axons in (**J**) CLEC12A^−/−^ mice compared to (**K**) WT mice at 7 dpi. (**C**,**F**,**I**,** L**) Statistical analysis: Mann-Whitney *U* test (*p ≤ 0.05: data are displayed as mean with standard deviation). (**A**,**B**,**D**,**E**,**G**,**H**,**J**,**K)** Scale bars = 250 μm; (**A**,**B**,**D**,**E**,**J**,**K**) inserts: scale bars = 50 μm; CLEC12A^−/−^ = ○, WT=▽.

The effect of CLEC12A deficiency on neuroinflammation and neuropathology was determined in mice infected with TMEV. Histology revealed an increased mononuclear cell infiltration of hippocampus in CLEC12A^−/−^ mice as compared to WT mice upon infection at 3 days post infection (dpi; *p* = 0.01; Fig. 1A–C). Likewise, a significant elevation of GFAP^+^ astrocytes (astrogliosis) was found in the brain of CLEC12A^−/−^ mice compared to WT mice at 3 dpi (*p* = 0.01, Fig. 1D–F). Indicative of termination of glial responses in knockout mice and ongoing neuroinflammation in WT mice, a gradual significant decline of GFAP^+^ astrocytes was found in the hippocampus of CLEC12A^−/−^ mice at 7 and 14 dpi (*p* = 0.01; *p* = 0.03 respectively, Fig. 1F).

Hippocampal damage was found in both groups following TMEV infection, characterized by a loss of NeuN^+^ mature neurons and increased number of damaged axons expressing β-APP. Densitometry revealed a significantly higher number of NeuN^+^ cells in the pyramidal layer of CLEC12A^−/−^ compared to WT mice at 7 dpi (*p* = 0.05, Fig. 1G–I). Moreover, a significant lower number of β-APP^+^ damaged axons was found in TMEV-infected CLEC12A^−/−^ mice at 7 dpi (*p* = 0.04, Fig. 1J–L). As expected, non-infected CLEC12A^−/−^ and WT mice showed no inflammation or hippocampal damage at any time point. Data show that intact CLEC12A signaling reduces neuroinflammation in C57BL/6 mice following TMEV infection. Increased inflammatory responses in CLEC12A^−/−^ mice did not lead to additional damage of hippocampal neurons, but even improved neuronal and axonal integrity.

**Fig. 2:**
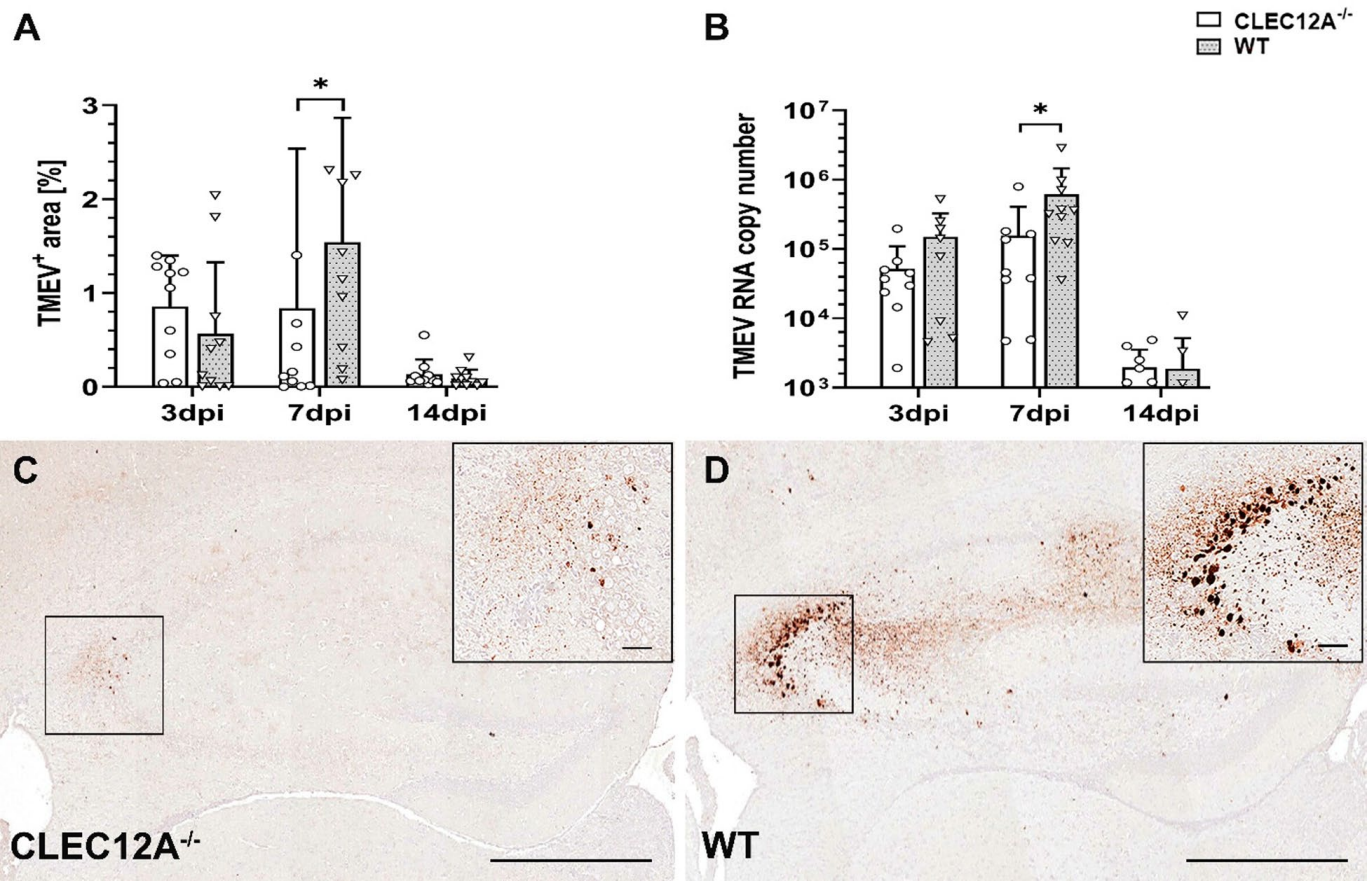
Theiler’s murine encephalomyelitis virus (TMEV) quantification in the brain. (**A**) TMEV antigen positive area labelled by immunohistochemistry. (**B**) TMEV mRNA measurement by reverse transcriptase quantitative polymerase chain reaction. Representative images (immunohistochemistry) showing TMEV antigen in the hippocampus of (**C**) CLEC12A^-/-^ and (**D**) wild type (WT) mice. (**A**,** B**) Statistical analysis: Mann-Whitney *U* test (*p ≤ 0.05: data are displayed as mean with standard deviation). (**C**,** D**) Scale bars = 250 μm; inserts: scale bars = 50 μm. dpi: days post infection; CLEC12A^-/-^ = ○, WT=▽.

TMEV exhibits a tropism for pyramidal cells of the hippocampus^13^. Accordingly, immunohistochemistry revealed a preferential infection of hippocampal neurons in CLEC12A^−/−^ and WT mice. Spatiotemporal analysis showed a significantly reduced TMEV load in the brain of CLEC12A^−/−^ mice compared to WT mice at 7 dpi (*p* = 0.04, Fig. 2A,C,D). In addition, RT-qPCR showed significantly reduced TMEV RNA levels in the brain of CLEC12A^−/−^ mice as compared to WT mice at 7 dpi (*p* = 0.02, Fig. 2B), suggesting that CLEC12A deficiency accelerates virus elimination. TMEV was not detected in the brain of non-infected mice from CLEC12A deficient and WT group.

In summary, CLEC12A deficiency leads to enhanced viral clearance from the brain, which is associated with preserved neuronal and axonal integrity in the hippocampus.

### CLEC12A deficiency is associated with improved T cell recruitment and pro-inflammatory responses in the brain

**Fig. 3:**
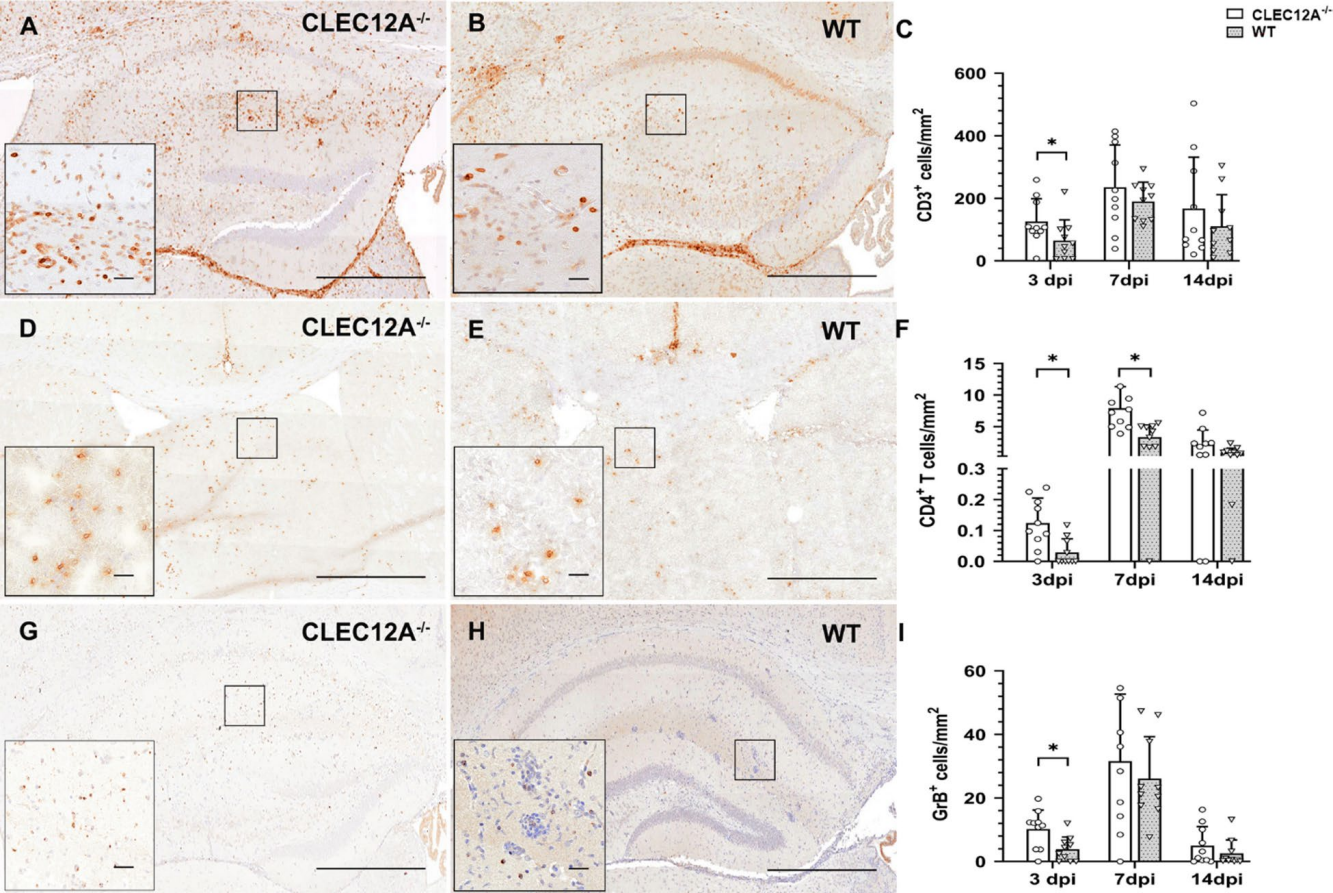
Phenotyping of inflammatory cells in the brain following Theiler’s murine encephalomyelitis virus (TMEV) infection. (**A–C**) Quantification of CD3^+^ T cells in the hippocampus. Representative images (immunohistochemistry) showing increased numbers of CD3^+^ T cells in (**A**) CLEC12A^-/-^ compared to (**B**) wild type (WT) mice at 3 days post infection (dpi). (**D–E**) CD4^+^ T cells in the cerebrum. Representative images (immunohistochemistry) showing increased numbers of CD4^+^ T cells in (**D**) CLEC12A^-/-^ compared to (**E**) WT mice at 3 and 7 dpi. (**G–I**) Quantification of granzyme B^+^ effector cells in the hippocampus. Representative images (immunohistochemistry) showing increased numbers of granzyme B^+^ cells in (**G**) CLEC12A^-/-^ compared to (**H**) WT mice at 3 dpi. (**C**,** F**,**I**) Statistical analysis: Mann-Whitney *U* test (*p ≤ 0.05: data are displayed as mean with standard deviation). (**A**,** B**,**D**,**E**,**G**,**H**) Scale bars = 250 μm; (**A**,**B**,**D**,**E**,**G**,**H**) inserts: scale bars = 20 μm; CLEC12A^-/-^ = ○, WT=▽.

Immunohistochemistry revealed a significantly increased infiltration of CD3^+^ T cells (*p* = 0.04, Fig. 3A–C) in the brain of CLEC12A^−/−^ mice as compared to WT mice at 3 dpi. CD45R^+^ B cells and CD107b^+^ macrophages/microglia did not show significant differences between groups at any time point (Supplementary Fig. S1B,C). As compared to CD8^+^ T cells, CD4^+^ T cells showed a relatively higher infiltration in animals lacking CLEC12A at 3 (*p* = 0.01) and 7 dpi (*p* = 0.01; Fig. 3D–F; Supplementary Fig.S1A). Granzyme B^+^ effector cells were significantly increased in the brain in response to neurotropic virus infection in CLEC12A deficient mice compared to WT mice at 3 dpi (*p* = 0.02, Fig. 3G–I). Non-infected animals from both groups showed no leukocytic infiltration to the brain at any time point. Data revealed that intact CLEC12A signaling in C57BL/6 mice negatively affects effector T cell sequestration in the brain during viral encephalitis.

**Fig. 4:**
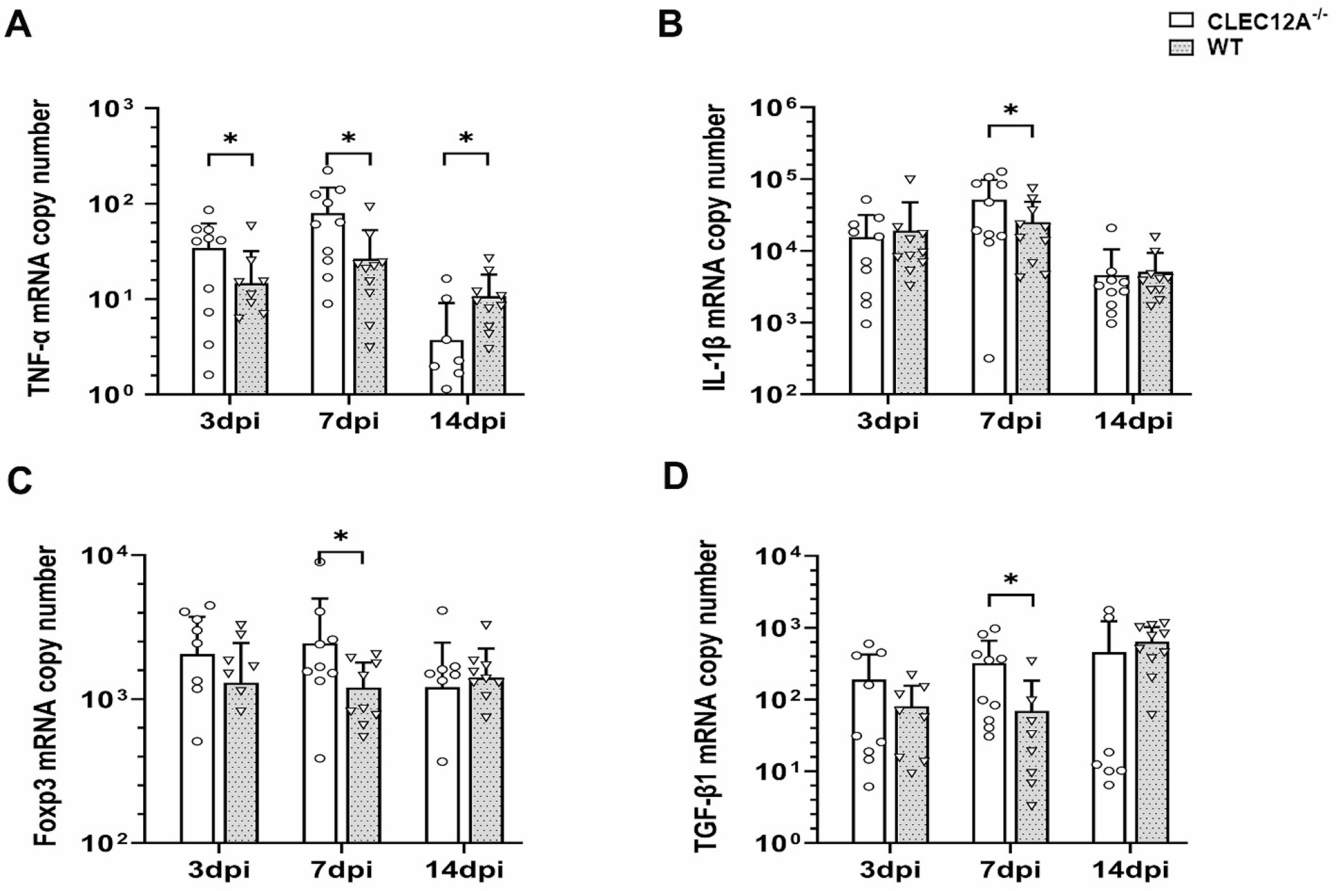
Pro and anti-inflammatory cytokine expression in the brain of Theiler’s murine encephalomyelitis virus (TMEV) infected mice. (**A**) Quantification of tumor necrosis factor (TNF)-α and (**B**) interleukin (IL)−1β, (**C**) *forkhead box protein P3* (*Foxp3*) and (**D**) *transforming growth factor-β1* (*TGF-β1*) in CLEC12A^-/-^ and wild type (WT) mice by reverse transcriptase quantitative polymerase chain reaction. (**A**–**D**) Statistical analysis: Mann-Whitney *U* test (*p ≤ 0.05: data are displayed as mean with standard deviation). dpi: days post infection; CLEC12A^-/-^ = ○, WT=▽.

To characterize the participating cytokine environment in the CNS, RT-qPCR was performed. Results revealed an enhanced *TNF-α* mRNA expression in the brain of CLEC12A^−/−^ mice as compared to WT mice at 3 dpi (*p* = 0.03) and 7 dpi (*p* = 0.01, Fig. 4A). In addition, immunohistochemistry revealed an increased TNF-α protein expression within the hippocampus of CLEC12A^−/−^ mice at 3 dpi (*p* = 0.05, Supplementary Fig. S3A-C). At 14 dpi, dynamics shifted and WT mice showed a statistical trend towards an increase of *TNF-α* mRNA expression compared to CLEC12A^−/−^ mice (*p* = 0.05, Fig. 4A), likely related to prolonged virus infection in WT mice. *IL-1β* transcription was significantly higher in CLEC12A^−/−^ mice compared to WT mice at 7 dpi (*p* = 0.05, Fig. 4B).

Regulatory T cells account for the termination of inflammation in order to maintain tissue homeostasis and avoid virus-mediated immunopathology^4^. At 7 dpi, mRNA expression level of *Foxp3*, a marker for regulatory T cells, was significantly elevated in the brain of CLEC12A^−/−^ mice as compared to WT mice (*p* = 0.05, Fig. 4C). In addition, mRNA copy numbers of *TGF-β1* in the brain of CLEC12A^−/−^ mice differed significantly to WT mice at 7 dpi, indicating the onset of compensatory immune responses (*p* = 0.01, Fig. 4D). Expression of other investigated cytokines (IFN-α, IFN-β, IFN-γ, IL-1α, IL-4, IL-5, IL-6) in the brain did not differ significantly between CLEC12A^−/−^ and WT mice (Supplementary Fig.S2A–G).

In conclusion, enhanced T cell recruitment and pro-inflammatory cytokine responses in the brain of CLEC12A^−/−^ mice at the early infection stage suggest a robust immune response, while intact CLEC12A signaling in WT mice is accompanied by impaired virus control following TMEV infection. Diminished pro-inflammatory cytokine responses in the brain of CLEC12A^−/−^ mice at later stages (14 dpi) is likely a consequence of reduced viral load and accelerated termination of neuroinflammation in mice lacking the inhibitory CLR.

### Early enhancement of neuroinflammation in CLEC12A deficient mice correlates with the activation of antigen presenting cells

**Fig. 5:**
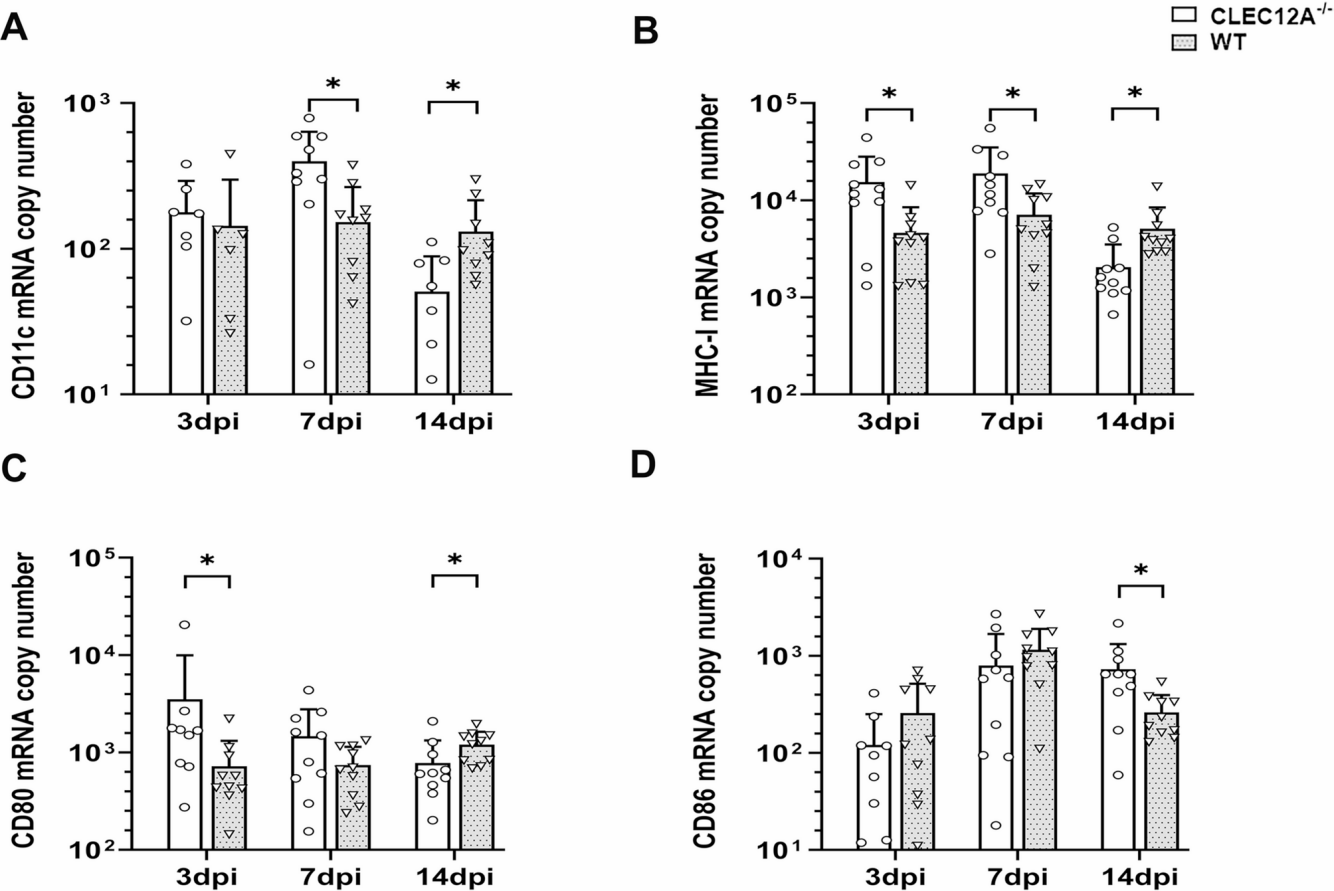
*CD11c*, *MHC-I CD80*, and *CD86* expression in the brain of Theiler’s murine encephalomyelitis virus (TMEV) infected CLEC12A^-/-^ and wild type (WT) mice. Quantification of (**A**) *CD11c*, (**B**) *MHC-I*, (**C**) *CD80*, and (**D**) *CD86* mRNA copy numbers by reverse transcriptase quantitative polymerase chain reaction. (**A–D**) Statistical analysis: Mann-Whitney *U* test (*p ≤ 0.05: data are displayed as mean with standard deviation). dpi: days post infection; CLEC12A^-/-^ = ○, WT=▽.

To investigate whether CLEC12A deficiency was associated with alterations in the expression of genes related to antigen presentation and co-stimulation, the mRNA expression of *CD11c*, *CD80*, *CD86*, and *major histocompatibility complex class I* (*MHC-I*) were quantified in the brain by RT-qPCR. Results showed that *CD11c* mRNA transcripts were significantly higher in the brain of CLEC12A^−/−^ mice at 7 dpi compared to WT mice following TMEV infection (*p* = 0.01, Fig. 5A). Similarly, significantly increased numbers of CD11c^+^ cells were found in the hippocampus of CLEC12A^−/−^ mice at 7 dpi by immunohistochemistry (*p* = 0.02, Supplementary Fig.S3D–F). *MHC-I* mRNA quantities were significantly increased in the brain of CLEC12A^−/−^ mice at 3 and 7 dpi compared to WT mice (*p* = 0.02; *p* = 0.04 respectively, Fig. 5B). Immunohistochemistry showed significantly increased numbers of MHC-I^+^ cells in the hippocampus of CLEC12A^−/−^ mice at 3 and 7 dpi (*p* = 0.05; *p* = 0.01; Supplementary Fig.S3G–I). *CD80* mRNA expression in the brain of CLEC12A^−/−^ mice significantly differed from WT mice at 3 dpi (*p* = 0.04, Fig. 5C). At 14 dpi, the dynamics shifted and *CD11c*, *CD80* and *MHC-I* mRNA expression were significantly higher in the brain of WT mice as compared to CLEC12A^−/−^ mice (*p* = 0.02; *p* = 0.02; *p* = 0.01 respectively, Fig. 5A–C), likely related to prolonged inflammation in the brain of infected WT mice. *CD86* mRNA levels in the brain of CLEC12A^−/−^ differed significantly compared to WT mice at 14 dpi (*p* = 0.02, Fig. 5D).

Conclusively, enhanced expression of *CD11c* together with co-stimulatory molecules in CLEC12A^−/−^ mice may be indicative of an increased antigen presenting capacity, thus enhancing the ability to trigger T cell responses in the brain. In addition, CLEC12A deficiency increases *MHC-I* expression following TMEV infection, likely linked to an increased presentation of viral antigens to cytotoxic T cells.

### CLEC12A deficiency enhances peripheral T cell responses following neurotropic virus infection

**Fig. 6:**
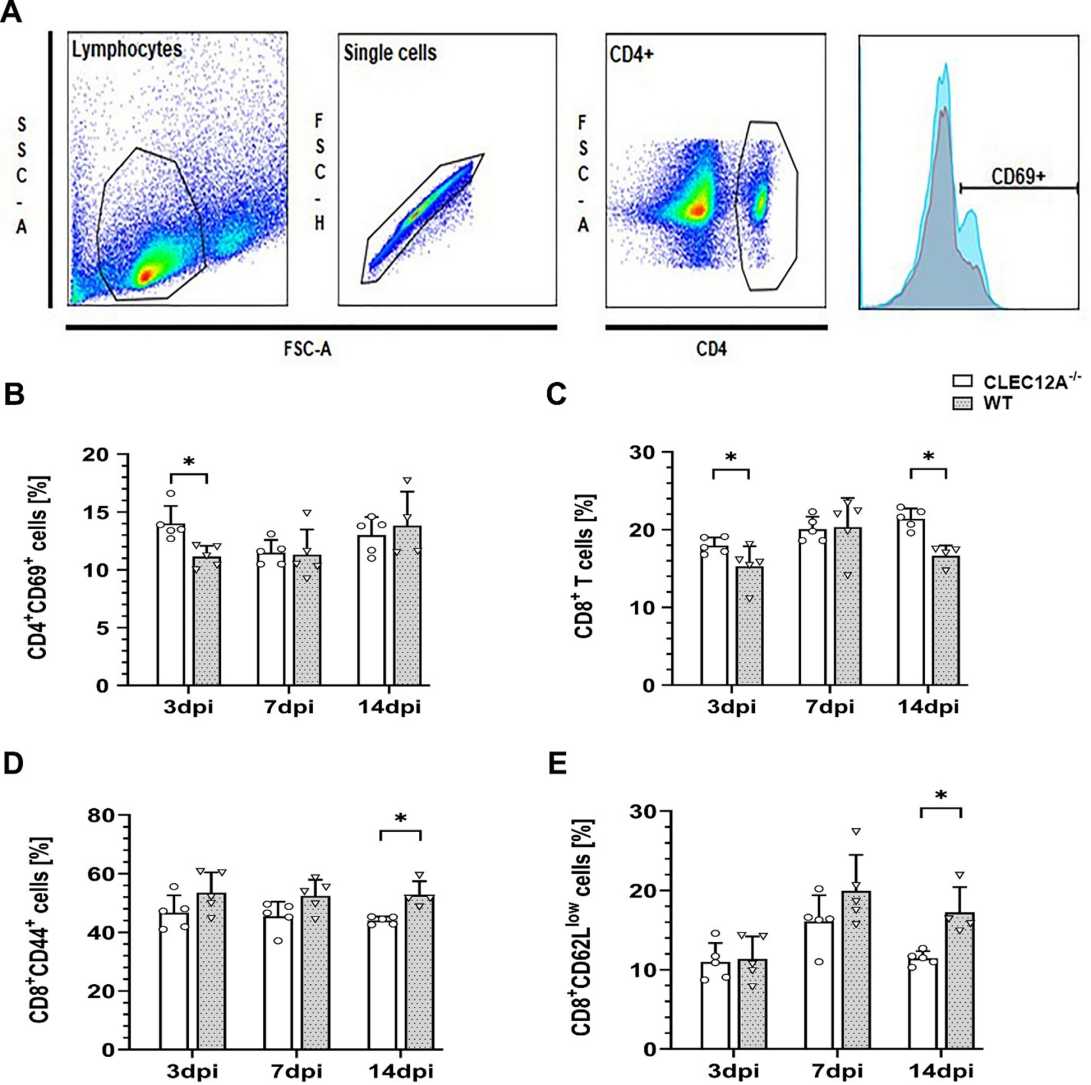
Phenotyping of splenic T cells by flow cytometry following Theiler’s murine encephalomyelitis virus (TMEV) infection of CLEC12A^−/−^ and wild type (WT) mice. (**A**) Gating strategy for the detection of activation markers on CD4^+^ T cells. Cells were first gated on lymphocytes, next on single cells, and next on CD4^+^ T cells. The frequency of activated CD4^+^ T cells among the CD4^+^ T cell population was determined (exemplified by representative histogram plots for CD69 expression). A comparable hierarchical gating strategy was applied to determine the frequency of activated CD8^+^ T cells.(**B**) Percentage of CD4^+^CD69^+^ T cells (related to all CD4^+^ T cells), (**C**) percentage of CD8^+^ T cells (related to total lymphocytes), (**D**) percentage of CD8^+^CD44^+^ T cells (related to all CD8^+^ T cells), and (**E**) percentage of CD8^+^CD62L^low^ T cells (related to all CD8^+^ T cells) in the spleen of TMEV-infected mice. (**B-D**) Statistical analysis: Mann-Whitney *U* test (*p ≤ 0.05: data are displayed as mean with standard deviation). dpi: days post infection; CLEC12A^−/−^ = ○, WT=▽.

To characterize the dynamics of CD4^+^ and CD8^+^ T cell responses of the peripheral immune system upon the neurotropic virus infection, flow cytometry of spleen tissue was performed. Timely priming of naïve T cells by antigen presenting cells decisively determines antiviral immune responses in the TMEV model^39,40^. Analysis revealed significantly higher frequencies of activated CD4^+^ T cells expressing the early activation marker CD69 at 3 dpi (*p* = 0.01, Fig. 6A,B) and an increased fraction of CD8^+^ cytotoxic T cells at 3 and 14 dpi (*p* = 0.06, *p* = 0.02; Fig. 6C) in the spleens of CLEC12A^−/−^ mice as compared to WT mice upon TMEV infection. At 14 dpi, an increased percentage of CD8^+^ cells expressing CD44 and reduced CD62L levels was found in WT mice (*p* = 0.02, *p* = 0.02 respectively Fig. 6D,E) indicative of prolonged T cell activation and sustained inflammation in mice with intact CLEC12A signaling.

Results suggest an early enhanced priming of peripheral T cell responses in CLEC12A^−/−^ mice upon TMEV infection, resulting in an improved antiviral immunity during viral encephalitis.

## Discussion

CLEC12A is a myeloid inhibitory CLR mostly expressed by antigen presenting cells^30^. This study demonstrates that CLEC12A signaling plays a critical role in modulating antiviral immune responses. During TMEV infection, CLEC12A deficiency is associated with enhanced peripheral T cell activation, faster recruitment of activated lymphocytes to the brain, and accelerated viral clearance. Despite heightened immune responses, CLEC12A^−/−^ mice exhibit reduced neuronal and axonal damage with preserved hippocampal integrity, suggesting that CLEC12A acts as a modulator of the initiated inflammation at the cost of prolonged viral infection leading to increased neurodegeneration upon neurotropic picornavirus infection.

CLEC12A^−/−^ mice show accelerated T cell recruitment to the brain with increased numbers of granzyme B^+^ effector cells and enhanced *MHC-I* expression during the early stage of TMEV infection (3 dpi). In addition, increased numbers of CD4^+^CD69^+^ T cells and CD8^+^ cytotoxic T cells in the spleen indicate strengthened peripheral T cell responses in CLEC12A^−/−^ mice following TMEV infection. A rapid induction of T cell responses, particularly CD8^+^ cytotoxic T cell responses, is critical for combating acute infection with intracellular pathogens^41^. In TMEV infection, the disease outcome depends decisively on the genetic background of mice^42^. While SJL and FVB mice develop insufficient antiviral immunity, which leads to chronic CNS infection and demyelinating disease in the spinal cord, C57BL/6 mice clear the virus after acute encephalitis and are resistant to persistent infection^13^. Protective immunity and TMEV clearance in C57BL/6 mice are linked to the H-2D MHC class I locus and mediated by robust MHC class I-restricted cytotoxic T cell responses^43–46^. CD8^+^ T cells of C57BL/6 mice recognize TMEV capsid epitopes and lyse virus-infected cells through granzyme B release^47–50^. The central role of early cytotoxicity has also been shown in transgenic FVB mice expressing the D^b^ class I molecule, which promotes robust antiviral cytotoxicity and TMEV elimination^45,51^. Similarly, the adoptive transfer of TMEV-specific cytotoxic T cells prevents virus persistence and demyelinating disease in SJL mice, while C57BL/6 mice lacking CD8^+^ T cells develop chronic infection and myelin loss. Moreover, the ablation of CD8^+^ T cells or the MHC class I component β_2_-microglobulin in C57BL/6 mice results in reduced antiviral cytotoxicity^26,52,53^.

An increased expression of *CD11c* and *CD80* was found in the brain of infected CLEC12A^−/−^ mice. CD11c^+^ antigen presenting cells have been shown to be critical for the priming of CD8^+^ T cells and early viral clearance in TMEV-infected C57BL/6 mice^54^. Besides CD8^+^ T cells, CD4^+^ T cells are required for protective immunity in TMEV infection, since CD4 deficient C57BL/6 mice exhibit reduced antiviral responses and impaired viral elimination^26,55,56^. CD4^+^ T cells support antiviral cytotoxicity by licensing of dendritic cells for antigen presentation to CD8^+^ T cells in peripheral lymphoid organs^41^. In addition, the co-stimulatory molecule CD80 (also known as B7-1) is expressed on activated innate immune cells and promotes cytotoxic T cell differentiation in viral infections^57^.

In the present study, the mRNA expression of *IL-1β* and *TNF-α* was upregulated in the brain of CLEC12A^−/−^ mice upon infection. Both pro-inflammatory cytokines are primarily produced by innate immune cells and enhance host defense mechanisms in infectious disorders^58–60^. The comparatively low expression of *IL-1β* and *TNF-α* in wild type C57BL/6 mice might be related to the inhibitory effect of intact CLEC12A signaling upon pro-inflammatory pathways^30,61,62^. IL-1β activates antiviral immune responses and IL-1 receptor knockout C57BL/6 mice have been shown to develop TMEV persistence^63–65^. However, excessive IL-1β administration also exerts pathogenic effects by elevating pathogenic Th17 responses, rendering C57BL/6 mice susceptible to demyelinating disease. Similarly, TNF-α exhibits dual functions in the TMEV infection model, likely depending on the disease phase and expression level. TNF-α has been shown to enhance protective immunity, and its genetic ablation in C57BL/6 mice delays healing of hippocampus following TMEV infection^66^. However, TNF-α also contributes to limbic hyperexcitability during the acute phase and Th1-mediated immunopathology during chronic TMEV infection, demonstrating the importance of a balanced cytokine response in the CNS following neurotropic virus infection to prevent immune mediated tissue damage^67,68^.

Although cytotoxicity and pro-inflammatory responses contribute to effective antiviral immunity in TMEV infection, T cells that target the hippocampus have been observed also to induce neuronal injury to acutely infected C57BL/6 mice^49,69^. Moreover, enhanced MHC class I-restricted cytotoxicity contributes to blood-brain barrier disruption in C57BL/6 mice following TMEV infection^70–72^. Interestingly, despite an enhancement of cytotoxic responses and accelerated virus elimination in CLEC12A^−/−^ mice, hippocampal pathology was even reduced following infection in the present study. Data suggests the presence of compensatory mechanisms that terminate inflammation and maintain tissue homeostasis. In this regard, an enhanced expression of *TGF-β1* and *Foxp3* was detected in the brain of CLEC12A^−/−^ mice following TMEV infection. Foxp3^+^ regulatory T cells mitigate immunopathology by inhibiting encephalitogenic T cells in primary autoimmune disorders (e.g. experimental autoimmune encephalitis (EAE)) and promote neural tissue repair following CNS injury^4^. Noteworthy, although regulatory T cells can contribute to disease exacerbation and viral persistence in neurotropic virus infection because of their immunosuppressive properties, no adverse effects on antiviral immunity during acute TMEV infection has been demonstrated in C57BL/6 mice in previous studies. Neither the adoptive transfer or expansion of regulatory T cells nor the depletion of Foxp3^+^ T cells impacts TMEV replication in the brain of C57BL/6 background mice^73–75^. The limited effect of regulatory T cells to suppress antiviral immunity in C57BL/6 mice is likely mediated by the dominant effect of cytotoxic CD8^+^ T cells during acute TMEV infection^76^. The modulatory cytokine TGFβ facilitates the brain recruitment and function of regulatory T cells. In addition, it has been shown to dampen inflammation and promote tissue recovery in a variety of neurologic disorders, such as EAE, stroke and toxoplasmosis^77–79^. In TMEV-infected C57BL/6 mice, TGFβ expression in neurons correlates with hippocampal damage, suggestive of a protective mechanism against neurodegeneration^6^. Moreover, TGFβ administration in SJL mice has been shown to reduce TMEV-induced immunopathology and demyelination^80^.

CLEC12A deficiency enhances peripheral immune responses and neuroinflammation, but does not exacerbate hippocampal injury following TMEV infection. On the contrary, an improved integrity of NeuN^+^ pyramidal neurons and less β-APP^+^ damaged axons were found in CLEC12A^−/−^ mice, indicating that lack of CLEC12A elicits a protective immune response without amplifying virus-induced immunopathology in the TMEV infection model. Results of the present study are in line with the observed inhibitory effects of CLEC12A signaling on pro-inflammatory signaling and T cell responses in sterile inflammation, immune-mediated colitis, rheumatoid arthritis, and mycobacterial infection models^32–35,81^. In contrast, CLEC12A signaling exhibited protective antiviral effects by increasing type I interferon responses in experimental LCMV and influenza virus infection of mice^38^. Moreover, CLEC12A^−/−^ mice develop modulated cytotoxic T cell responses following *Plasmodium* infection, thus conferring protection from experimental cerebral malaria^36^. This indicates that CLEC12A signaling exhibits divergent roles in infectious disorders and may elicit protective and detrimental effects depending on the disease setting. The cause for different functions of CLEC12A signaling in viral disease models remains speculative but might be attributed to the activation of heterologous signaling pathways by diverse exogenous and endogenous ligands. Comparably, the CLR Mincle (also known as CLEC4E) has been shown to exhibit negative or positive effects on immunity caused by coupling to an activating ITAM or to an inhibitory ITAM depending on the nature of ligand^82^.

Similar effects of CLEC12A found in this study have been observed for the inhibitory CLR DCIR in neurotropic virus infection. Our previous study revealed that DCIR^−/−^ mice show an ameliorated hippocampal integrity and accelerated viral elimination with increased cytotoxic CD8^+^ T cell responses following TMEV infection^29^. Moreover, activation of the adaptor protein CARD9 that delivers signals through ITAM-coupled activating CLRs has been shown to reduce the loss of NeuN^+^ neurons and β-APP^+^ damaged axons in the hippocampus of TMEV infected mice^83^.

While our results clearly demonstrate a substantial role of CLEC12A in regulating the immune response to TMEV, the distinct CLEC12A ligand sensed during TMEV infection yet remains to be elucidated. To date, CLEC12A has been primarily reported to recognize crystalline structures such as monosodium urate (MSU) crystals and hemozoin^34,36^. As CLEC12A was shown to sense MSU crystals released from dying cells during sterile inflammation, which may also be a potential mode of action of CLEC12A during the inflammatory response in TMEV infection. However, CLEC12A was also reported to bind to yet unidentified endogenous proteins from dead cells, rendering the recognition of dead cell-derived proteins by CLEC12A in the context of TMEV infection possible as well^28,34^. Moreover, a recent study showed that CLEC12A binds to mycolic acid, a major component of mycobacterial cell walls, and thereby modulates the host immune response^32^. Since the ligand-binding sites of CLEC12A for mycolic acid and MSU overlap with each other, this may enable the recognition of different types of ligands by CLEC12A, including viral ligands^84^. Hence, the sensing of TMEV capsid proteins by CLEC12A cannot be excluded. However, there was no indication of direct CLEC12A-virus interactions in other viral infections, such as murine LCMV or influenza infection, rendering TMEV recognition by CLEC12A during TMEV infection unlikely^38^. Instead, it is more probable that CLEC12A signaling may be triggered by host cells destroyed by TMEV or the induced antiviral immune response and/or the release of DAMPs from dying cells, such as MSU.

In conclusion, our findings highlight CLEC12A as a regulator of antiviral immunity and neuroinflammation in the TMEV infection model, suggesting that targeting inhibitory CLRs could represent an approach to enhance protective immunity while minimizing virus-induced neuropathology. However, given the diverse roles of CLEC12A across different infectious and inflammatory diseases, further studies are clearly required to elucidate the precise mechanisms governing its function in the CNS and its potential as a therapeutic target in viral encephalitis and other neuroinflammatory conditions.

## Materials and methods

### Experimental design

CLEC12A^−/−^ mice were generated at the Institute of Clinical Chemistry and Pathobiochemistry of the Technical University of Munich^34^ and backcrossed on C57BL/6 background over more than ten generations at the Institute of Biochemistry of the University of Veterinary Medicine Hannover^85^. Female, 5-week-old CLEC12A^−/−^ mice (*n* = 30, body weight 14.0–18.5 g) and C57BL/6 mice (WT; *n* = 30, body weight 13.4–19.4 g) were infected intracerebrally with TMEV DA. Non-infected CLEC12A^−/−^ (*n* = 5, body weight 22.7–24.6 g) and C57BL/6 mice (*n* = 5, body weight 20.5–22.4 g) were used as controls. All these animals were kept in ventilated cages and provided *ad libitum* water and rodent feed under controlled conditions (humidity 50–60%, temperature 22–24 °C, light and dark cycle for 12 h each). TMEV DA propagation for intracerebral infection was performed on BHK-21 cells with a MOI of 1.0. The virus titer was determined via plaque assays on L-cells as described previously^29^. Mice were anaesthetized with an intraperitoneal injection of medetomidine (1 mg/kg, Domitor) and ketamine (100 mg/kg) and inoculated into the right cerebral hemisphere with TMEV DA in a total volume of 20 µl DMEM (Biochrom GmbH, Berlin, Germany) supplemented with 2% FCS (PAA Laboratories GmbH, Pasching, Austria) and 50 µg/kg gentamicin (Sigma Aldrich Chemie GmbH, Taufkirchen, Germany) as described^76^. Weekly clinical examinations included body weight recording, appearance, behavior and gait evaluation^76^. None of the animals showed any signs of distress, significant pain, motor dysfunction or seizures during the infection period. Euthanasia was performed at predefined time points (3, 7 and 14 dpi) according to the ethical guidelines. To minimize suffering, euthanasia was performed with an overdose of medetomidine (1 mg/kg) and ketamine (200 mg/kg) and tissues were taken at necropsy^29^. A transverse cut of the brain was made at the optic chiasm level and the rostral part was snap-frozen and stored at −80 °C for RNA isolation and immunostaining (CD4 and CD8 markers)^76,86^. The caudal part of the cerebrum was fixed in 10% formalin for 24 h, embedded in paraffin wax and sectioned at the hippocampal level of 2–3 μm thickness (Bregma − 1.46 to −1.82) for histology (hematoxylin-eosin staining) and immunohistochemistry^87^.

### Immunohistochemistry

Antibodies were used to label neurons (neuronal nuclei, NeuN), axons (β-amyloid precursor protein, β-APP), astrocytes (glial fibrillary acidic protein, GFAP), TMEV capsid protein VP-1, macrophages/microglia (CD107b), T cells (CD3, CD4, CD8), B cells (CD45R), cytotoxic T cells/natural killer cells (granzyme B), antigen presenting cells (CD11c, MHC-I) and tumor necrosis factor-α (TNF-α) are listed in Supplementary Table S1. Immunohistochemistry of brain sections was performed as described previously^88–90^. In brief, slides were deparaffinized (except for frozen sections, CD4 and CD8) and endogenous peroxidases were blocked by 0.5% hydrogen peroxide (H_2_O_2_) in 85% ethanol. For CD4 and CD8 staining, phosphate buffer saline (PBS) was used instead of ethanol. Heat-induced epitope retrieval with citrate buffer (pH 6.0–6.5) for 30 min in a microwave oven was performed for NeuN, β-APP, CD107b, CD3, CD45R, granzyme B, TNF-α, CD11c and MHC-I labeling. Blocking of non-specific bindings was achieved by goat serum (NeuN, β-APP, GFAP, TMEV, CD3, granzyme B, TNF-α, CD11c, MHC-I) and rabbit serum (CD107b, CD4, CD8). PBS with 1% bovine serum albumin (BSA) was used to dilute primary antibodies, followed by overnight incubation at 4 °C. Biotinylated goat anti-rabbit (for GFAP, TMEV, CD3, CD11c, MHC class I), rabbit anti-rat (for CD107b, CD4, CD8) and goat anti-mouse (for NeuN, β-APP, TNF-α) IgG antibodies were used as secondary antibodies. To visualize the binding of antibodies, avidin-biotin-peroxidase complex (ABC) diluted in PBS along with 3, 3’-diaminobenzidine (DAB) as chromogen in PBS and 0.5% H_2_O_2_ was used. Mayer’s hematoxylin was used as a counterstain^88,90^.

### Digital image analysis

Analyses of HE-stained and immuno-stained slides of CLEC12A^−/−^ mice (*n* = 30) and WT mice (*n* = 30) were performed on three serial brain sections per animal. Slides were scanned using an Olympus VS200 Digital Slide Scanner (Olympus Deutschland, GmbH) at 20x magnification with brightfield imaging mode for the quantitative evaluation of the hippocampus and cerebrum. Open-source software QuPath (version 0.5.1) was used for densitometric analysis^91^. Cell quantification of HE-stained hippocampal sections was achieved by automated cell detection feature, based on nuclear staining using the hematoxylin OD channel. Results were normalized to hippocampal area and expressed as cells per mm^2^. Random tree classifier was interactively trained for pyramidal NeuN^+^ neurons, hippocampal GFAP^+^ astrocytes, CD107b^+^ macrophages/microglia, CD45R^+^ B cells, TMEV^+^ and TNF-α expression to distinguish DAB-stained positive cells from the artifacts. Staining artifacts from the regions of interest (ROIs) were removed using channel specific thresholds^92^. Results were expressed as percentage of positively stained area of each marker to the total area of respective ROI. CD3^+^ T cells, β-APP^+^ axons, granzyme B^+^ effector cells, CD11c^+^ cells and MHC-I^+^ cells in the hippocampus were manually quantified and expressed as count per mm^2^ of hippocampus. Likewise, quantification of CD4^+^ helper T cells and CD8^+^ cytotoxic T cells in the entire cross section of cerebrum was performed manually.

### Ribonucleic acid isolation and reverse transcription

RNA was isolated from snap-frozen cerebral tissues as described^29^. The purity and quantity of isolated RNA were measured using a Multiskan™ GO microplate spectrophotometer (Thermo Fisher Scientific, Braunschweig, Germany), µDrop™ plate, SkanIt™ version 7.0.2, Microplate Reader version 7.0.2.5. An equal amount of isolated RNA was reversely transcribed into cDNA using Omniscript^®^ Reverse Transcription Kit (Qiagen, Hilden, Germany), RNaseOUT™ Recombinant Ribonuclease Inhibitor (Invitrogen™) and random primers (Promega Corporation, Walldorf, Germany).

### Reverse transcription-quantitative polymerase chain reaction (RT-qPCR)

The viral load (TMEV) in the cerebrum and mRNA expression levels of interleukin (IL)−1α, IL-1β, IL-4, IL-5, IL-6, Foxp3, tumor necrosis factor (TNF)-α, interferon (IFN)-α, IFN-β, IFN-γ, transforming growth factor (TGF)-β1, CD11c, CD80, CD86 and MHC-1 were quantified by RT-qPCR as described^29^ using AriaMx Real-Time PCR System (Agilent Technologies Deutschland GmbH, Waldbronn, Germany) software version 2.1 and Brilliant III Ultra-Fast SYBR^®^ Green qPCR Mastermix (Agilent Technologies Deutschland GmbH, Waldbronn, Germany). Primer details are listed in Supplementary Table S2. The standard curve method with serial dilutions was used to quantify the copy numbers. Melting curve analysis was used to control the specificity of each reaction^86^.

The data were normalized using three reference genes (hypoxanthine-guanine phosphoribosyl transferase (HPRT), glyceraldehyde 3-phosphate dehydrogenase (GAPDH) and β-actin with geNorm software version 3.4^93^.

### Flow cytometric analysis of murine splenocytes

Spleens were removed and immediately flushed mechanically with a syringe and 1 × PBS to a single cell suspension. Subsequently erythrocytes were lysed using RBC lysis buffer (10% 100 mM Tris–HCl [Tris-(hydroxymethyl)-aminomethanhydrochloride], 90% 160 mM NH_4_Cl [ammonium chloride], Carl Roth, Karlsruhe, Germany). Afterwards, cells were incubated with rat-anti CD16/32 monoclonal antibody (1:100) to block the Fc gamma receptor and therefore to avoid unspecific binding. Cell solutions were stained with following monoclonal anti-mouse antibodies: CD4-FITC, CD4-PE, CD4-PerCP-Cy5.5, CD8a-FITC, CD8a-PE, CD8a-APC, CD62L-PE, CD62L-PE-Cy7, CD69-APC and CD44-APC. Details for all flow cytometry antibodies are listed in Supplementary Table S3. For fixation, cells were incubated with 1% paraformaldehyde (PFA, Carl Roth, Karlsruhe, Germany). Flow cytometry was performed at the Attune NxT cytometer (Thermo Fisher Scientific, Waltham, MA, USA). Data analysis was conducted with FlowJo software (version 10, FloJo LLC, Ashland, OR, USA)^94^.

### Statistical analysis

To compare both study groups (CLEC12A^−/−^ and WT), a non-parametric, Mann–Whitney *U*-tests, for non-normal distributed data, was applied using IBM SPSS^®^ software, Windows version 26^95^. *P*-value ≤ 0.05 was considered statistically significant. Graphs were plotted using the GraphPad Prism software version 9.0.0.

## Supplementary Information

Below is the link to the electronic supplementary material.

Supplementary Material 1

Supplementary Material 2

Supplementary Material 3

Supplementary Material 4

## Data Availability

The data presented in this study are available on request from the corresponding author.
