# Supplementary material for: CLEC12A signaling represses protective immune responses and contributes to hippocampal pathology in neurotropic picornavirus infection
Source: Sci Rep. 2025 Nov 10;15:39354. doi: 10.1038/s41598-025-27365-3 (PMC12603091; doi:10.1038/s41598-025-27365-3)
Supplement: Supplementary file 1 — Supplementary Material 1 [file 41598_2025_27365_MOESM1_ESM.pdf]

Supplementary information

CLEC12A signaling represses protective immune responses and contributes to hippocampal pathology in neurotropic picornavirus infection

MK Ameen[1,2‡], M Stoff[1‡], S Pavasutthipaisit[1], T Ebbecke[2,3,4], M Ciurkiewicz[1], T Störk[1], J Ruland[5,6,7,8,9], B Lepenies[2,3,4,10*], A Beineke[1,2*†]

[1]Department of Pathology, University of Veterinary Medicine Hannover, Germany, [2]Center for Systems Neuroscience, Hannover, Germany, [3]Institute for Immunology, University of Veterinary Medicine Hannover, Germany, [4]Research Center for Emerging Infections and Zoonoses, University of Veterinary Medicine Hannover, Germany, [5]Institute of Clinical Chemistry and Pathobiochemistry, School of Medicine, Technical University of Munich, 80333 Munich, Germany, [6]Center for Translational Cancer Research (TranslaTUM), 81675 Munich, Germany, [7]German Cancer Consortium (DKTK), Partner Site Munich, 80336 Munich, Germany, [8]German Research Center (DKFZ), 69120 Heidelberg, Germany, [9]German Center for Infection Research (DZIF), Partner Site Munich, 17493 Greifswald, Germany, [10]Chair of Biochemistry and Chemistry, Veterinary Faculty, Ludwig-Maximilians-Universität München, Munich, Germany

‡ These contributed equally first to this work

* These contributed equally last to this work

[†]Corresponding author: andreas.beineke@tiho-hannover.de

Supplementary Figure legends:

Supplementary Figure S1. Profiling of immune cells in the brain following Theiler's murine encephalomyelitis virus (TMEV) infection. Quantification of cerebral (**A**) CD4$^+$ T cells and (**B**) CD8$^+$ T cells and of hippocampal (**C**) CD45R$^+$ B cells and (**D**) CD107b$^+$ macrophages/microglia in CLEC12A$^{-/-}$ and WT mice by immunohistochemistry. Mann-Whitney *U* test (*$p \leq 0.05$: data are displayed as mean with standard deviation). dpi: days post infection; CLEC12A$^{-/-}$ = ○, WT=▽.

Supplementary Figure S2. Quantification of cytokines expression in the brain of Theiler's murine encephalomyelitis virus (TMEV) infected mice. (**A**) IFN-α (**B**) IFN-β (**C**) IFN-γ (**D**) IL-1α (**E**) IL-4 and (**F**) IL-5 (**G**) and IL-6 mRNA expression in CLEC12A$^{-/-}$ and wild type (WT) mice by reverse transcriptase quantitative polymerase chain reaction. (**A-G**) Statistical analysis: Mann-Whitney U test (data are displayed as mean with standard deviation). dpi: days post infection; CLEC12A$^{-/-}$ = ○, WT=▽.

Supplementary Figure S3. Quantification of TNF-α, CD11c and MHC I expression in the brain by immunohistochemistry. (**A-C**) TNF-α expression. Representative images of the hippocampus showing increased TNF-α expression in (**A**) CLEC12A$^{-/-}$ compared to (**B**) wild type (WT) mice at 3 days post infection (dpi). (**D-E**) Quantification of CD11c$^+$ cells. Representative images of the hippocampus showing increased numbers of CD11c$^+$ cells in (**D**) CLEC12A$^{-/-}$ compared to (**E**) WT mice at 7 dpi. (**G-I**) Quantification of MHC I$^+$ cells. Representative images of the hippocampus showing increased numbers of MHC I$^+$ cells in (**G**) CLEC12A$^{-/-}$ compared to (**H**) WT mice at 3 dpi. (**C,F,I**) Statistical analysis: Mann-Whitney *U* test (*$p \leq 0.05$: data are displayed as mean with standard deviation). (**A,B,D,E,G,H**) Scale bars = 250 µm; (**A,B,D, E,G,H**) inserts: scale bars = 20 µm; individual values CLEC12A$^{-/-}$ = ○, WT=▽.

Supplementary Table S1. Details of antibodies used for immunohistochemistry

Primary antibody/dilution	Supplier (clone/catalogue number)	Pre-treatment	Blocking solution	Secondary antibody	Specificity
CD107b 1:200 (mc)	AbD Serotec (MCA2293)	20 minutes citrate buffer/MW	Rabbit	Rabbit anti-rat	Macrophages/microglia
CD3 1:500(pc)	DakoCytomation (A0452)	20 minutes citrate buffer/MW	Goat	Goat-anti-rabbit	T cells
CD4 1:2000 (mc)	BD Pharmingen, (550280)	-	Rabbit	Rabbit anti-rat	$CD4^+$ T cells
CD8 1:1000 (mc)	BD Pharmingen, (553038)	-	Rabbit	Rabbit anti-rat	$CD8^+$ T cells
CD45R 1:1000 (biotinylated mc)	BD Biosciences (553085)	20 minutes citrate buffer/MW	-	-	B cells
GFAP 1:1000 (pc)	DakoCytomation (Z0334)	-	Goat	Goat anti-rabbit	Astrocytes
Granzyme B 1:400 (pc)	Abcam (ab4059)	20 minutes citrate buffer/MW	Goat	Goat anti-rabbit	Effector T cells and NK cells
NeuN 1:1600 (mc)	Merck/Millipore (MAB377)	20 minutes citrate buffer/MW	Goat	Goat anti-mouse	Neurons
TMEV 1:2000 (pc)	Department of Pathology, University of Veterinary Medicine Hannover, Germany*	-	Goat	Goat anti-rabbit	TMEV capsid protein VP1
β-APP 1:2000 (mc)	Merck/Millipore (MAB348)	20 minutes citrate buffer/MW	Goat	Goat anti-mouse	Axonal damage
TNF-α 1:50 (mc)	Novus NBP2-34303)	20 minutes citrate buffer/MW	Goat	Goat anti-mouse	Inflammatory cytokines producing cells

CD11c 1:500 (pc)	Invitrogen (PA5-79537)	20 minutes citrate buffer/MW	Goat	Goat anti-rabbit	Activated microglia and infiltrating myeloid or dendritic cells
MHC-I 1:200 (pc)	Biorbyt (Orb5449)	20 minutes citrate buffer/MW	Goat	Goat anti-rabbit	Presentation of endogenous peptides to $CD8^+$ T cell

β-APP = beta-amyloid precursor protein; GFAP = glial fibrillary acidic protein; mc = monoclonal; MW = microwave oven; NeuN = neuronal nuclei; pc = polyclonal; TMEV = Theiler's murine encephalomyelitis virus; TNF-α = tumor necrosis factor-α; NK cells = natural killer cells; *Kummerfeld et al. [88]

Supplementary Table S2. Detail of primer sequences used for reverse transcriptase quantitative polymerase chain reaction

Gene	Accession No.	mRNA position	Primer direction	Primer sequence 5'→3'	PCR product size (bp)
HPRT	NM-013556.2	646-665<br>833-814	Forward<br>Reverse	GGA CCT CTC GAA GTG TTG GA<br>TCG TAT TTG CAG ATT CAA CT	188
GAPDH	NM-001289726.1	351-370<br>638-619	Forward<br>Reverse	GAG GCC GGT GCT GAG TAT GT<br>GGT GGC AGT GAT GGC ATG GA	288
β-actin	NM-007393.5	698-717<br>930-911	Forward<br>Reverse	GGC TAC AGC TTC ACC ACC AC<br>ATG CCA CAG GAT TCC ATA CC	233
TMEV DA	M-20301.1	1596-1615<br>1833-1814	Forward<br>Reverse	TGG TCG ACT CTG TGG TTA CG<br>GCC GGT CTT GCA AAG ATA GT	238
IFN-α	NM-010502.2	67-88<br>176-197	Forward<br>Reverse	GGA TGT GAC CTT CCT CAG ACT C<br>ACC TTC TCC TGC GGG AAT CCA A	131
IFN-β	NM-010510.1	154-178<br>229-208	Forward<br>Reverse	TGA ATG GAA AGA TCA ACC TCA CCTA<br>CTC TTC TGC ATC TTC TCC GTC A	76

IFN- γ	NM-008337.4	175-194<br>299-318	Forward<br>Reverse	CAC GGC ACA GTC ATT GAA AG<br>AAT CTG GCT CTG CAG GAT TT	144
IL-1α	NM-010554.4	294-313<br>472-453	Forward<br>Reverse	AAG CAA CGG GAA GAT TCT GA<br>TGA CAA ACT TCT GCC TGA CG	179
IL-1β	NM-008361.4	311-330<br>460-439	Forward<br>Reverse	AGC TAC CTG TGT CTT TCC CG<br>AGT GCA GTT GTC TAA TGG GAA C	150
IL-5	NM-.10558.1	104-123<br>283-264	Forward<br>Reverse	ATG GAG ATT CCC ATG AGC AC<br>CCC ACG GAC AGT TTG ATT CT	180
IL-6	NM-031168.2	250-269<br>425-404	Forward<br>Reverse	GTT CTC TGG GAA ATC GTG GA<br>CCA GAG GAA ATT TTC AAT AGG C	176
TNF-α	NM-013693.3	268-287<br>470-451	Forward<br>Reverse	GCC TCT TCT CAT TCC TGC TT<br>CAC TTG GTG GTT TGC TAC GA	203
TGF-β1	NM-011577.2	1719-1738<br>1901-1882	Forward<br>Reverse	TTG CTT CAG CTC CAC AGA GA<br>TGG TTG TAG AGG GCA AGG AC	183
Foxp3	NM-054039.2	801-821<br>889-869	Forward<br>Reverse	CCC AGG AAA GAC AGC AAC CTT<br>TTC TCA CAA CCA GGC CAC TTG	89
CD11c	NM-021334.3	2396-2417<br>2530-2509	Forward<br>Reverse	TGC CAG GAT GAC CTT AGT GTC G<br>CAG AGT GAC TGT GGT TCC GTA G	135
CD80	NM-001359898.1	287-309<br>426-405	Forward<br>Reverse	CCT CAA GTT TCC ATG TCC AAG GC<br>GAG GAG AGT TGT AAC GGC AAG G	140
CD86	NM-019388.3	837-860<br>983-962	Forward<br>Reverse	ACG TAT TGG AAG GAG ATT ACA GCT<br>TCT GTC AGC GTT ACT ATC CCG C	147
MHC-I	NM-001001892.2	344-365<br>449-428	Forward<br>Reverse	GGC AAT GAG CAG AGT TTC CGA G<br>CCA CTT CAC AGC CAG AGA TCA C	106

Accession No. = GenBank sequence accession number and version (National Center for Biotechnology Information, U.S. National Library of Medicine, Bethesda MD, USA), bp: base pairs, GAPDH = glyceraldehyde 3-phosphate dehydrogenase, HPRT = hypoxanthine-guanine phosphoribosyltransferase, Foxp3 = Forkhead box p3, IFN = interferon, IL = interleukin, TNF-α = tumor necrosis factor α, TGFβ1 = transforming growth factor β1.

Supplementary Table S3. **Detail of monoclonal antibodies used for flow cytometry**

Antibody and conjugate	Supplier (clone)	Dilution	Specificity
CD4-FITC	BD Biosciences, BD PharmingenTM, Heidelberg, Germany (clone GK1.5)	1:100	$CD4^+$ helper T cells
CD4-FITC	Thermo Fisher Scientific, Invitrogen, eBioscienceTM, Waltham, MA, USA (clone RM4-5)	1:100	$CD4^+$ helper T cells
CD4-PE	BD Biosciences, BD PharmingenTM, Heidelberg, Germany (clone GK1.5)	1:100	$CD4^+$ helper T cells
CD4-PerCP Cy5.5	Thermo Fisher Scientific, Invitrogen, eBioscienceTM, Waltham, MA, USA (clone RM4-5)	1:200	$CD4^+$ helper T cells
CD8a-FITC	Thermo Fisher Scientific, Invitrogen, eBioscienceTM, Waltham, MA, USA (clone 53-6.7)	1:100	$CD8^+$ cytotoxic T cells
CD8a-APC	BD Biosciences, BD PharmingenTM, Heidelberg, Germany, clone 53-6.7)	1:200	$CD8^+$ cytotoxic T cells
CD8a-PE	BD Biosciences, BD PharmingenTM, Heidelberg, Germany (clone 53-6.7)	1:200	$CD8^+$ cytotoxic T cells
CD44-APC	BD Biosciences, BD PharmingenTM, Heidelberg, Germany (clone IM7)	1:200	Activated T cells, memory T cells
CD62L-PE	BD Biosciences, BD PharmingenTM, Heidelberg, Germany (clone MEL-14)	1:200	T cell activation marker, downregulation upon T cell activation, L-selectin
CD62L-PE-Cy7	Thermo Fisher Scientific, Invitrogen, eBioscienceTM, Waltham, MA, USA (clone MEL-14)	1:200	T cell activation marker, downregulation upon T cell activation, L-selectin
CD69-APC	Thermo Fisher Scientific, Invitrogen, eBioscienceTM, Waltham, MA, USA (clone H1.2F3)	1:200	T cell activation marker, upregulation upon T cell activation

APC = allophycocyanin, Cy = cyanine dye, FITC = fluorescein isothiocyanate, PE = phycoerythrin, PerCP = peridinin chlorophyll protein complex
