# Supplementary figures and images for: CLEC12A signaling represses protective immune responses and contributes to hippocampal pathology in neurotropic picornavirus infection
Source: Sci Rep. 2025 Nov 10;15:39354. doi: 10.1038/s41598-025-27365-3 (PMC12603091; doi:10.1038/s41598-025-27365-3)

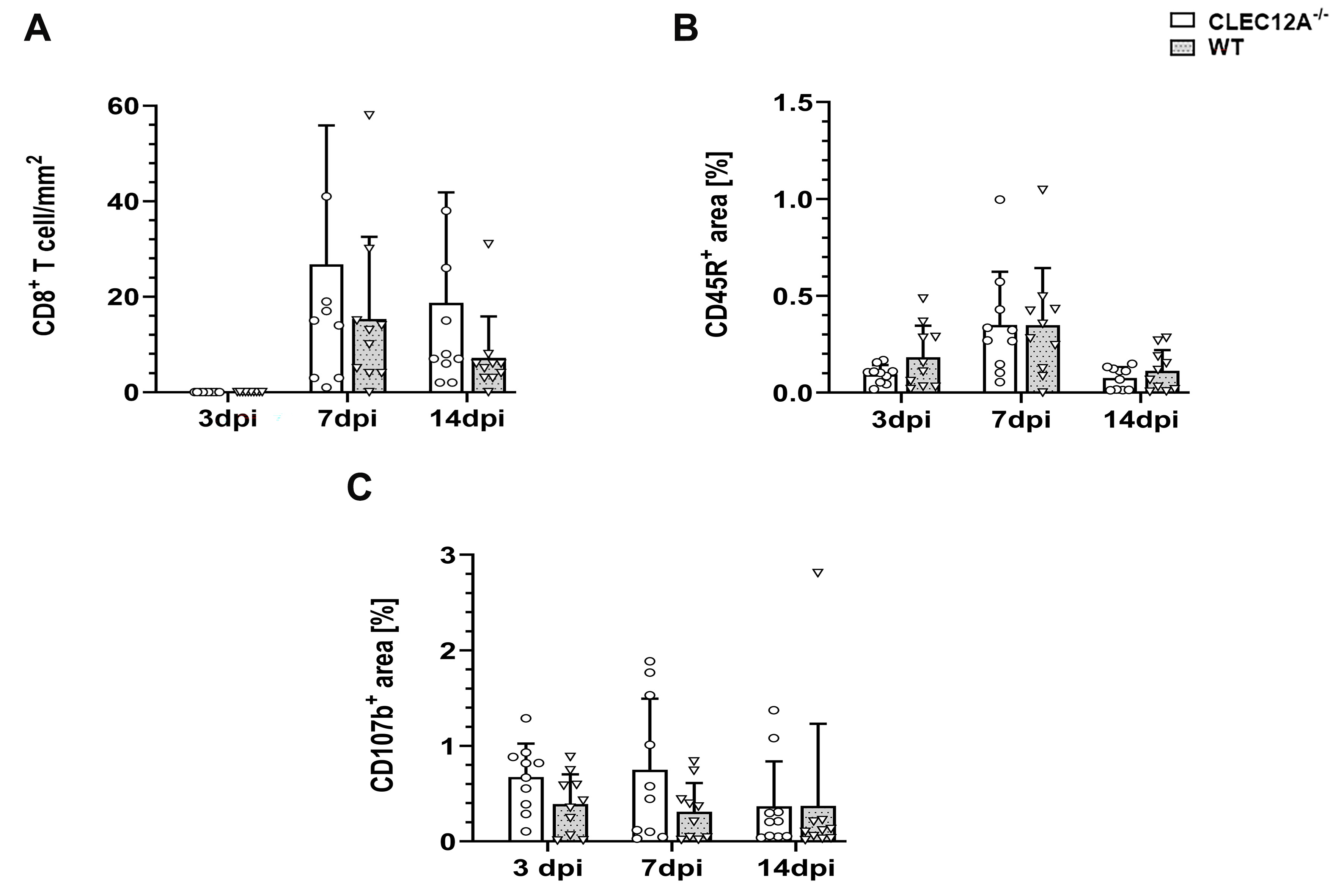

Supplement: Supplementary file 2 — Supplementary Material 2 [file 41598_2025_27365_MOESM2_ESM.jpg]

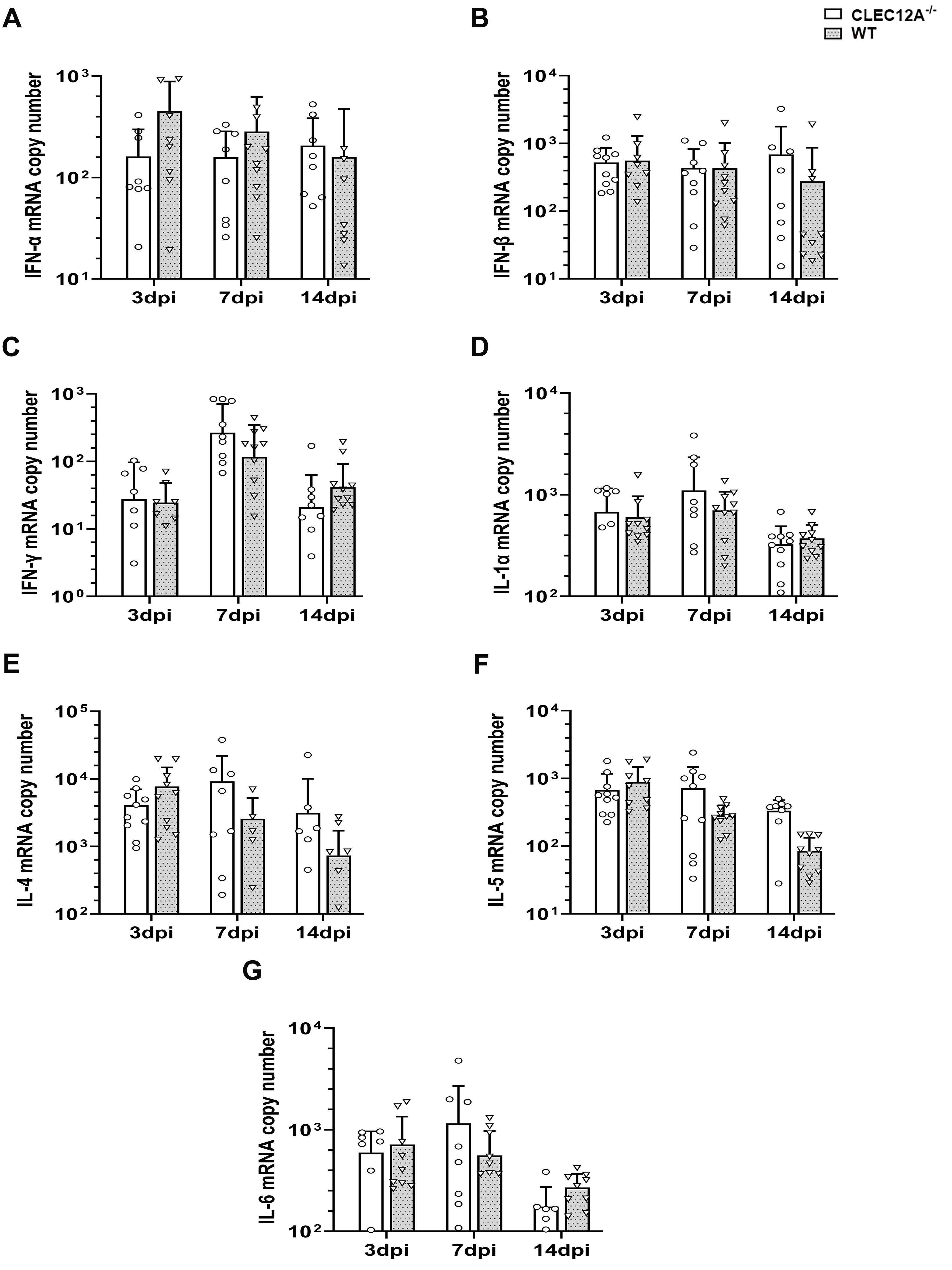

Supplement: Supplementary file 3 — Supplementary Material 3 [file 41598_2025_27365_MOESM3_ESM.jpg]
